# Prevalence of metabolic syndrome and its association with oral health: First results from the Kurdish cohort study

**DOI:** 10.1002/hsr2.1602

**Published:** 2023-10-12

**Authors:** Farhad Moradpour, Zahra Karimi, Zeinab Fatemi, Yousef Moradi, Mohammad Rastegar Khosravi, Azad Shokri, Mohammad Karimzadeh

**Affiliations:** ^1^ Social Determinants of Health Research Center, Research Institute for Health Development Kurdistan University of Medical Sciences Sanandaj Iran; ^2^ Vice Chancellor for Research and Technology Kurdistan University of Medical Sciences Sanandaj Iran; ^3^ Vice Chancellor for Health Affairs Kurdistan University of Medical Sciences Sanandaj Iran; ^4^ Department of Epidemiology and Biostatistics, Faculty of Medicine Kurdistan University of Medical Sciences Sanandaj Iran; ^5^ Department of Endodontics, School of Dentistry Kurdistan University of Medical Sciences Sanandaj Iran

**Keywords:** dental caries, DMF index, metabolic syndrome, oral health

## Abstract

**Aims:**

Investigate the association between oral and dental health (ODH) and metabolic syndrome (MetS) in adults aged 35–70 years.

**Methods:**

The study utilized data from the enrollment phase of Dehgolan prospective cohort study in the west of Iran. A cross‐sectional assessment was conducted on a total of 3996 participants, involving a comprehensive oral examination and the assessment of their oral hygiene behavior (ODH). MetS was defined according to the updated National Cholesterol Education Program Adult Treatment Panel III. Logistic regression used to calculate odds ratios (ORs) and 95% confidence intervals (CIs), adjusting for sociodemographic characteristics.

**Results:**

MetS was more prevalent among those who not daily brushing and flossing. Participants with missing teeth (MT) show higher prevalent of MetS. Being female, lower age, illiteracy, family history of diabetes, low physical activity, and salt at table were independently associated with increase odds of MetS (*p* < 0.05). Odds of MetS were significantly decreased with use flossing (OR = 0.75; CI = 0.60–0.93), decayed (OR = 0.83; CI = 0.72–0.97), filled (OR = 0.84; CI = 0.71–0.99), and increased with MT (OR = 1.45; CI = 1.16–1.81) as long as adjusted for ODH factors. When other potential confounder such as sociodemographic, personal and nutritional habits were adjusted, daily flossing was the only factor that still statistically decreased (OR = 0.79; CI = 0.62–0.99) the odds of MetS.

**Conclusion:**

Daily flossing was the only factor that independently associated with MetS. Relationship of other ODH factors with Mets were confounded by sociodemographic characteristics of the participants.

## 1 INTRODUCTION

In the current two decades, metabolic syndrome (MetS) are being increasingly recognized as the most common health problem affecting people of all ages.^1^ MetS is associated with several factors including insulin resistance, visceral adiposity, dyslipidemia, endothelial dysfunction, genetic susceptibility, elevated blood pressure (BP), and chronic inflammation.^2^

The presence of MetS in general population is responsible for one‐third of cardiovascular disease risk^3^ and around 6%–7% of all‐cause. mortality.^4^ The overall prevalence of MetS varied from 12.5% to 31.4% in the adult population worldwide.^5^ Specifically, in Kurdish population the prevalence of MetS is reported to be 22.1%.^6^

It's suggested that chronic inflammation associated with metabolic abnormality may leading to insulin resistance and MetS.^7^ Various studies reported that oral caries and MetS are interrelated and share common risk factors that include abdominal obesity, excess sugar intake, weight, age, smoking, alcohol consumption, income, and physical inactivity.^8^, ^9^ Some studies have pointed out the link between chronic periodontitis with chronic diseases such as dyslipidemia,^10^ diabetes,^11^ and MetS. Likewise, it has been shown that dental caries process directly or indirectly related to the metabolic conditions.

Since both MetS and dental caries are correlated with common factors such as inflammation, it is reasonable to surmise they share the same causal pathways. Dental caries is a long‐term and irreversible process, caused by lack of oral hygiene and past chronic infection and inflammation.^2^, ^12^ So, it can be used as a suitable proxy for oral and dental health (ODH).

Another Index that directly associated with dental caries is DMFT (decayed, missing, filled teeth), the most common index used to evaluate ODH is based on a person's experience of decay (from the past to present) and indicates the oral health status of a person throughout his life.^13^

However, the relationship between MetS and oral health remain controversial and limited studies^14^, ^15^ have investigated the relationship between oral health behaviors and MetS. The purpose of this study is to directly assess the relationship between DMFT with MetS and its component separately by taking into account the oral health behaviors.

## 2 MATERIAL AND METHODS

### 2.1 Study design and population

This study was used data of Dehgolan prospective cohort study (DehPCS) from individuals aged 35–70 years after enrollment phase. Study detail and rational has published elsewhere.^16^ Briefly, DehPCS is a part of prospective epidemiological research in Iran (PERSIAN) a combination of population‐based studies evaluating incidence, prevalence, mortality, and risk factors of common noncommunicable diseases. A total of 3996 permanent residents of Dehgolan town were selected. Subjected with severe illness unable to go through questioning process and/or those who were unwilling to participate were excluded. The DehPCS was gathered socioeconomic, self‐reported health status such as medical history, sign and symptoms, and biomarker assessment. The research was performed ethically according to the principles of the Declaration of Helsinki and was approved by the Ethics Committee of the Kurdistan University of Medical Sciences, Kurdistan, Iran (IR. MUK. REC.1400.323). The participants to be included signed the informed consent form.

### 2.2 Measurements and data collection

Data collection was carried out via a face‐to‐face interview by local trained interviewer who spoke Kurdish. First, participant were taken a sample of blood and urine after 8–12 h of fasting. Afterward, weight and height and waist circumference (WC) were measured by Seca scale and Stadiometer with accuracy of 0.1 cm. Then, body mass index (BMI) was categorized (body mass [kg]/height [m^2^]) as: normal weight ≤ 24.9, overweight = 25.0–29.9, and obese ≥ 30.0.

They were classified as “smoker” if had a history of smoking ≥ 100 cigarettes life time, ex‐smoker, or never smoker. Use of 200 mL of beer or 45 mL of other alcoholic beverage once a week for at least 6 month was considered as “alcohol user.” Illicit/illegal drug use including once a week for at least 6 months was classified as “use drugs.”

Self‐reported time on leisure, work, sport, and other activity during 1 week was registered by standard questionnaire. Metabolic equivalent task score per hour per day was calculated based on participant's weight, type, and duration of activities. Then physical activity (PhA) as an everyday practice was categorized into low (24–36.5), moderate (36.6–44.9), and vigorous (≥45). Education level was classified in the following four categories that is, illiterate (zero school years), 1–6 school years, 7–12 school years, and university (>12 school years).

Wealth index was used as a proxy for socioeconomic status of participants. It is a composite measure of household living standards, calculated using easy‐to‐collect data on family ownership of selected assets. We used principle correspondence analysis to calculate total wealth score of asset data. Then participants were ranked and distributed into three groups of poorest, middle, and richest.

Salt at table and added sugar were used as a marker of the poor dietary habits. We divided added sugar into the two categories of low (women <25 & men <38) and high (women≥ 25 & men ≥ 38).^17^

### 2.3 Definition of MetS

MetS is a cluster of risk factors that often occur together and increase the risk of developing various health conditions, including cardiovascular diseases and type 2 diabetes. We defined MetS according to the National Cholesterol Education Program Adult Treatment Panel III which was revised by American Heart Association/National Heart, Lung, and Blood Institute in 2005.^18^ To establish a positive diagnosis of the MetS, three out of five criteria have to be met, as follows: (1) elevated WC ≥ 102 cm in men and ≥88 cm in women, (2) elevated serum triglyceride (TG) ≥ 150 mg/dL and/or on drug treatment for elevated TG, (3) reduced high‐density lipoprotein‐cholesterol (HDL‐C) < 40 mg/dL in men and <50 mg/dL in women and/or on drug treatment for reduced HDL‐C, (4) elevated BP ≥ 130 mmHg systolic BP or 85 mmHg diastolic BP and/or on antihypertensive drug treatment in a patient with a history of hypertension, and (5) elevated fasting plasma glucose ≥ 100 mg/dL and or on drug treatment for elevated glucose. BP was measured using a standard protocol by trained nurses with the participants in sitting position twice in right arm after at least 15 min of resting before measuring. The mean of two measurements was used as Systolic and diastolic BP.

### 2.4 Oral health measurement

The oral cavity of all participant undertook a comprehensive clinical examination. The number of decayed teeth (DT), missing teeth (MT), and filled teeth (FT) was diagnosed according to WHO criteria using DMFT index. In line with standard guidelines published by WHO,^19^ participants with MT due to reason other than carious extraction such as trauma were excluded from the analysis. Teeth with restoration on one surface and caries on the other were counted as decayed. DMFT score was estimated for the total sample according to the following criteria: 0 = free from any form of dental decay, missing or FT and ≥1 = having one or more of dental decay, missing or FT. Subsequently, subjects were divided into three groups of low (<9), moderate (9–13.9), and high (≥14) DMFT. Participants were also asked about their ODH behavior such as daily brushing, flossing, and use of mouthwash.

### 2.5 Statistical analysis

Prevalence of MetS was defined across the different oral heath variable as the number of participants with positive criteria for MetS in nominator divided by all participants. The characteristics of participants were reported by count (proportion) and were compared between those with and without MetS using *χ* ^2^ test. Logistic regression model was used for calculate odds ratio (OR) with 95% confidence intervals (CI) for MetS, elevated WC, reduced HDL‐C, elevated BP, and elevated fast blood sugar (FBS). All analyses were conducted by using Stata software, version 16.0 (Stata Corp). The *p* < 0.05 was considered a statistically significant, clinically relevant level of association.

## 3 RESULTS

In total, 3992 participates were enrolled in the study. A total of 56.26% of participants were females. Mean ages of female and male participants were 47.98 ± 8.91 and 48.78 ± 8.91, respectively. Approximately, 91% of participants were married, 31% were illiterate, and 13% had university education. At least 75% of participants had a BMI of 25 or higher, and 46% comorbid with hypertension or dyslipidemia.

### 3.1 Prevalence of MetS

Table 1 presents prevalence of MetS across sociodemographic characteristics of the participants in the DehPCS. There were 1362 (34.62%, CI: 32.66–35.60) patient with MetS. Prevalence of MetS was higher among women than man with ratio of 2.10. The prevalence was increased by about 25% in the ages over 60 and decreases with education and socioeconomic levels by approximately 31% and 7%, respectively. MetS decreased in current smoker, illicit/illegal drug user, and alcohol user by 44.47%, 27.72%, and 33.35%, respectively. Proportion of MetS linearly increased with reporting family history of diabetes in first degree and both first and second degree. Vigorous physically active participants have shown less prevalence of MetS. In terms of healthy diet behaviors, participants who use salt at table present higher MetS. Inversely, those who use high amount of add sugar have shown less proportion of MetS.

**Table 1:** Prevalence of MetS based on the socioeconomic characteristics, personal and nutritional habit of the participants in DehPCS.

	Without MetS	With MetS	Total	*p* Value
Gender				
Male	1378 (78.92)	368 (21.08)	1746	<0.001
Female	1252 (55.74)	994 (44.26)	2246	
Age groups				
35–45	1328 (74.44)	456 (25.56)	1784	<0.001
46–60	1064 (61.75)	659 (38.25)	1723	
>60	238 (49.07)	247 (50.93)	485	
Marital status				
Married	2459 (67.06)	1208 (32.94)	3667	<0.001
Divorced/widow/single	171 (52.62)	154 (47.38)	325	
Education years				
Illiterate	625 (50.16)	621 (49.84)	1246	<0.001
1–5	742 (66.79)	369 (33.21)	1111	
6–12	843 (75.20)	278 (24.80)	1121	
University	420 (81.71)	94 (18.29)	514	
Economic status				
Poor	958 (62.41)	577 (37.59)	1535	<0.001
Middle	750 (65.39)	397 (34.61)	1147	
Reach	903 (70.11)	385 (29.89)	1288	
BMI				
Normal weight	883 (89.28)	106 (10.72)	989	<0.001
Overweight	1145 (67.16)	560 (32.84)	1705	
Obese	591 (45.96)	695 (54.04)	1286	
Cigarette smoking				
No smoker	1917 (63.39)	1107 (36.61)	3024	<0.001
Ex‐smoker	208 (63.80)	118 (36.20)	326	
Smoker	478 (79.67)	122 (20.33)	600	
Illicit/illegal drug use				
No	2269 (64.79)	1233 (35.21)	3502	<0.001
Yes	334 (74.55)	114 (25.45)	448	
Alcohol use				
No	2236 (64.44)	1234 (35.56)	3470	<0.001
Yes	367 (76.30)	114 (23.70)	481	
Family history of diabetes				
No	1663 (68.27)	773 (31.73)	2436	<0.001
Second degree	296 (68.84)	134 (31.16)	430	
First degree	505 (61.36)	318 (38.64)	823	
First and second degree	150 (53.00)	133 (47.00)	283	
Physical activity (MET‐hour/daily)				
Low	1023 (63.19)	596 (36.81)	1619	<0.001
Moderate	1138 (63.68)	649 (36.32)	1787	
Vigorous	444 (80.29)	109 (19.71)	553	
Number of meals per day				
≤3	925 (64.46)	510 (35.54)	1435	0.157
4	1346 (67.30)	654 (32.70)	2000	
5–6	349 (64.27)	194 (35.73)	543	
Salt at table				
No	1219 (69.50)	535 (30.50)	1754	<0.001
Sometime	733 (67.50)	353 (32.50)	1086	
Yes	668 (58.70)	470 (41.30)	1138	
Added sugar				
Low	993 (61.41)	624 (38.59)	1617	<0.001
High	1637 (68.93)	738 (31.07)	2375	
Comorbidity				
No	1390 (87.20)	204 (12.80)	1594	<0.001
Dyslipidemia	945 (61.24)	598 (38.76)	1543	
Hypertension	173 (54.06)	147 (45.94)	320	
Both	122 (22.80)	413 (77.20)	535	

Abbreviations: BMI, body mass index; DehPCS, Dehgolan prospective cohort study; MET, metabolic equivalent task; MetS, metabolic syndrome.

Table 2 shows prevalence of MetS based on ODH factors. MetS was more prevalent in those who not daily brushing and flossing their teeth, by ratio of 1.14 and 1.35, respectively. Participants with denture use experience higher percentage of MetS (40.52, CI: 37.89–43.19). Of the 1439 participants with DMFT < 14, there were 431(29.95%, CI: 27.64–32.37) MetS, while more than one‐third of participants with DMFT ≥ 14 (36.64%, CI: 34.79–38.54) had MetS. Participants with DT and FT have shown lower values of MetS with prevalence of 32.13% (30.28–34.03) and 30.00% (27.76–32.34), respectively. However, MetS proportions across ordered MT groups were linearly increased (*p* trend < 0.001).

**Table 2:** Prevalence of MetS based on ODH factors of the participants in DehPCS.

	Without MetS	With MetS	Total	*p* Value
Daily brushing				
No	1083 (63.26)	629 (36.74)	1712	0.003
Yes	1526 (67.70)	728 (32.30)	2254	
Use moth wash				
No	2529 (65.86)	1311 (34.14)	3840	0.582
Yes	80 (63.49)	46 (36.51)	126	
Use flossing				
No	2222 (64.56)	1220 (35.44)	3442	<0.001
Yes	387 (73.85)	137 (26.15)	524	
Has denture				
No	1825 (68.92)	823 (31.08)	2648	<0.001
Yes	784 (59.48)	534 (40.52)	1318	
DMFT				
Low <9	444 (69.92)	191 (30.08)	635	<0.001
Moderate 9–13.9	564 (70.15)	240 (29.85)	804	
High ≥14	1601 (63.36)	926 (36.64)	2527	
DT				
No	993 (62.65)	592 (37.35)	1585	0.001
Yes	1616 (67.87)	765 (32.13)	2381	
FT				
No	1531 (63.11)	895 (36.89)	2426	<0.001
Yes	1078 (70.00)	462 (30.00)	1540	
MT				
Low >5	756 (72.83)	282 (27.17)	1038	<0.001
Moderate 6–15	932 (65.27)	496 (34.73)	1428	
High ≥16	921 (61.40)	579 (38.60)	1500	

Abbreviations: DehPCS, Dehgolan prospective cohort study; DMFT, decayed, missing, filled teeth; MetS, metabolic syndrome; ODH, oral and dental health.

### 3.2 Risk factor associated to the MetS and its components

Table 3 presents model 1 of crude and adjusted OR for MetS and its component according to the ODH factors. Among all participants, daily brushing, moderate and high MT were significantly increased adjusted odds of high WC by 46%, 58%, and 68%, respectively. Daily flossing and DT were decreased odds of WC by 18% and 15%, independently of other oral and dental factors. Those who have FT had a lower odd of developing high BP. People with moderate and high MT were approximately 1.5 and 2 times likely to develop hypertension. Adjusted odds of elevated FBS were decreased significantly by 18% among those with DT. Like hypertension, elevated FBS was directly affected by moderate and high MT. They increase probability of elevated FBS about 38% and 45%, respectively. Daily brushing significantly increased adjusted odds of HDL by 24%, but having DT decreased HDL by 19% independently of other dental factors.

**Table 3:** OR for MetS and its components according to the oral and dental correlates of participants in DehPCS.

	Elevated waist C		Elevated BP		Elevated FBS		Reduced HDL		MetS	
	Crud OR (CI)	Adjusted^a^ OR (CI)	Crud OR (CI)	Adjusted^a^ OR (CI)	Crud OR (CI)	Adjusted^a^ OR (CI)	Crud OR (CI)	Adjusted^a^ OR (CI)	Crud OR (CI)	Adjusted^a^ OR (CI)
Daily brush										
Yes (Ref: no)	1.14 (1.01–1.30)	1.46 (1.24–1.73)	0.51 (0.45–0.59)	0.89 (0.74–1.08)	0.71 (0.62–0.82)	0.94 (0.76–1.14)	1.34 (1.18–1.53)	1.24 (1.01–1.42)	0.82 (0.72–0.94)	1.12 (0.94–1.34)
*p* Value	0.044	<0.001	<0.001	0.251	<0.001	0.555	<0.001	0.014	0.004	0.211
Use moth wash										
Yes (Ref: no)	1.19 (0.82–1.72)		0.93 (0.62–1.40)		1.14 (0.77–1.69)		0.94 (0.66–1.45)		1.11 (0.77–1.60)	
*p* Value	0.355		0.738		0.517		0.744		0.582	
Use flossing										
Yes (Ref: no)	0.81 (0.67–0.97)	0.82 (0.67–0.99)	0.54 (0.43–0.69)	0.87 (0.68–1.13)	0.62 (0.53–0.83)	0.81 (0.63–1.03)	1.28 (1.06–1.55)	1.13 (0.94–1.40)	0.64 (0.52–0.79)	0.75(0.60–0.93)
*p* Value	0.024	0.046	<0.001	0.306	<0.001	0.093	0.009	0.183	<0.001	0.014
DT										
Yes (Ref: no)	0.90 (0.79–1.02)	0.85 (0.73–0.98)	0.64 (0.56–0.74)	0.85 (0.72–1.00)	0.74 (0.64–0.85)	0.82 (0.69–0.97)	0.98 (0.86–1.12)	0.81 (0.70–0.94)	0.79 (0.69–9.91)	0.83 (0.72–0.97)
*p* Value	0.102	0.029	<0.001	0.055	<0.001	0.019	0.777	0.005	0.001	0.020
FT										
Yes (Ref: no)	0.98 (0.86–1.12)		0.47 (0.40–0.55)	0.67 (0.55–0.81)	0.72 (0.62–0.84)	0.89 (0.74–1.07)	1.21 (1.07–1.28)	0.98 (0.84–1.15)	0.73 (0.64–0.84)	0.84 (0.71–0.99)
*p* Value	0.827		<0.001	<0.001	<0.001	0.231	0.003	0.810	<0.001	0.040
MT (Ref: low >5)										
Moderate 6–15	1.53 (1.30–1.80)	1.58 (1.34–1.87)	1.62 (1.32–1.98)	1.53 (1.24–1.89)	1.41 (1.16–1.71)	1.38 (1.13–1.68)	1.06 (0.90–1.24)	1.11 (0.94–1.31)	1.43 (1.20–1.70)	1.40 (1.17–1.68)
High ≥16	1.39 (1.1–1.63)	1.62 (1.32–1.99)	2.95 (2.42–3.58)	2.00 (1.56–2.56)	1.81 (1.50–1.19)	1.45 (1.13–1.85)	0.76 (0.65–0.90)	0.83 (0.67–1.03)	1.68 (1.42–2.00)	1.45 (1.16–1.81)
*p* Value	<0.001	<0.001	<0.001	<0.001	0.001, <0.001	0.001, 0.003	0.495, 0.001	0.202, 0.085	<0.001	<0.001

Abbreviations: BP, blood pressure; CI, confidence interval; DehPCS, Dehgolan prospective cohort study; FBS, fast blood sugar; HDL, high‐density lipoprotein; MetS, metabolic syndrome; OR, odds ratio.

^a^ Adjusted for daily brush; use moth wash; use flossing; DT; FT; and MT.

Table 4 exhibit model 2 of crud and adjusted ORs for MetS according to the demographic, socioeconomic, and selected oral and dental factors. Being female increased odds of MetS proximately 2.7 times, independently of other factors. Adjusted odds of MetS directly increased across age groups and, inversely decreased with number of education years. Individual with positive family history of diabetes have more chance to develop MetS. Reported DM in first and both first‐ and second‐degree relatives increased adjusted odds of MetS by 35% and 90%, respectively. People with vigorous PhA may decreased probability of MetS by 28% than those who have low PhA.

**Table 4:** OR for MetS according to the sociodemographic, ODH factors, personal and nutritional habits in participants of DehPCS.

	Crud OR (CI)	*p* Value	Adjusted^a^ OR (CI)	*p* Value
Gender (Ref: female)				
Male	0.34 (0.29–0.39)	<0.001	0.38 (0.31–0.48)	<0.001
Age groups^1^, ^4^, ^15^, ^20^, ^21^, ^22^, ^23^, ^24^, ^25^, ^26^, ^27^				
46–60	1.80 (1.56–2.08)	<0.001	1.82 (1.51–2.20)	<0.001
>60	3.02 (2.46–3.72)	<0.001	3.16 (2.35–4.25)	<0.001
Marital status (Ref: married)				
Single	1.83 (0.46–0.52)	<0.001	0.92 (0.68–1.25)	0.597
Education years (Ref: illiterate)				
1–5	0.50 (0.42–0.59)	<0.001	0.79 (0.65–0.97)	0.027
6–12	0.50 (0.42–0.59)	<0.001	0.70 (0.55–0.91)	0.007
University	0.22 (0.17–0.29)	<0.001	0.52 (0.36–0.75)	<0.001
Economic status (Ref: poor)				
Middle	0.90 (0.75–1.03)	0.113	1.09 (0.91–1.30)	0.361
Reach	0.91 (0.60–0.83)	<0.001	1.08 (0.89–1.31)	0.448
Cigarette smoking (Ref: no)				
Ex‐smoker	0.98 (0.77–1.24)	0.88	1.15 (0.87–1.52)	0.336
Smoker	0.58 (0.54–0.62)	<0.001	0.74 (0.55–1.01)	0.061
Illicit/illegal drug use (Ref: no)				
Yes	0.62 (0.51–0.78)	<0.001	1.27 (0.93–1.71)	0.127
Alcohol use (Ref: no)				
Yes	0.56 (0.45– 0.70)	<0.001	1.11 (0.84–1.47)	0.452
Family history of diabetes (Ref: no)				
Second degree	0.97 (0.78–1.21)	0.81	1.08 (0.85–1.37)	0.524
First degree	1.35 (1.15–1.59)	<0.001	1.37 (1.14–1.63)	0.001
First and second degree	1.90 (1.48–2.44)	<0.001	1.95 (1.49–2.56)	<0.001
MET‐hour (daily) (Ref: low)				
Moderate	0.98 (0.85–1.12)	0.76	0.89 (0.77–1.05)	0.166
Vigorous	0.42 (0.33–0.53)	<0.001	0.72 (0.55–0.93)	0.012
Number of meals per day (Ref: ≤3)				
4	0.88 (0.76–1.01)	0.083	0.99 (0.85–1.17)	0.924
5–6	1.01 (0.82–1.24)	0.938	1.16 (0.89–1.51)	0.276
Salt at table (Ref: no)				
Sometime	1.10 (0.93–1.29)	0.263	1.02 (0.85–1.21)	0.842
Yes	1.60 (1.37–1.87)	<0.001	1.36 (1.14–1.32)	<0.001
Added sugar (Ref: low)				
High	0.72 (0.63–0.82)	<0.001	0.80 (0.68–0.95)	0.012
Daily brush (Ref: no)				‐
Yes	0.82 (0.72–0.94)	0.004	1.04 (0.85–1.28)	0.682
Use moth wash (Ref: no)				
Yes	1.11 (0.77–1.60)	0.582		
Use flossing (Ref: no)				‐
Yes	0.64 (0.52–0.79)	<0.001	0.79 (0.62–0.99)	0.049
Has denture (Ref: no)			‐	‐
Yes	1.51 (1.32–1.73)	<0.001	‐	‐
DMFT	1.02 (1.01–1.03)	<0.001	‐	‐
DT (Ref: no)				‐
Yes	0.79 (0.69–9.91)	0.001	1.02 (0.86–1.21)	0.220
FT (Ref: no)				
Yes	0.73 (0.64–0.84)	<0.001	0.94 (0.77–1.14)	0.520
MT (Ref: Low >5)				‐
Moderate 6–15	1.43 (1.20–1.70)	<0.001	1.05 (0.86–1.28)	0.630
High ≥16	1.68 (1.42–2.00)	<0.001	0.84 (0.65–1.09)	0.190

Abbreviations: CI, confidence interval; DehPCS, Dehgolan prospective cohort study; DMFT, decayed, missing, filled teeth; MET, metabolic equivalent task; MetS, metabolic syndrome; ODH, oral and dental health; OR, odds ratio.

^a^ Adjusted for gender; age groups; marital status; education years; economic status; illicit/illegal drug use; alcohol use; family history of diabetes; MET‐hour; number of meals per day; salt at table; added sugar; daily brush; use moth wash; use flossing; has denture; DT; FT; and MT.

Adjusted odds of MetS were increased by 36% in people who use salt than those never uses salt at table. However, excessive added sugar intake was inversely related to the MetS (adjusted OR = 0.8, CI = 0.68–0.95).

In the univariable analysis daily brushing, flossing, DT, and FT were inversely related to the MetS and MT directly increased odds of MetS. But in the presence of demographic and socioeconomic factors, multivariable analysis revealed none of them related to the MetS, except for daily flossing which decreased odds of MetS by 21% (OR = 0.79, CI = 0.62–0.99).

## 4 DISCUSSION

The prevalence of MetS among 3992 participants was 34.62%. According to the results, the MetS prevalence in this study was higher than the national estimate of 23.8% in 2018^28^ and 30.4% in 2019,^29^ and less than national studies which reported the prevalence of MetS as 38.3% in 2021^30^ and 36.9% in 2015^31^ in Iran. Similar studies with the same age groups, Ravansar in Kermanshah (33.82%),^32^ and Kashan (35.9%)^24^ reported equal prevalence of MetS. Studies in Southeast Iran, Zahedan (21.0%),^33^ Rural Population in Fasa (24.2%),^34^ Sistan and Baluchestan Province (18.3%),^29^ Tehran (27%)^35^ were low. The prevalence was higher in northern Iran (42.3%)^36^ and Bushehr Province (57.8%),^29^ and among in food insecurity population (45.5%).^37^ Also, the prevalence varies throughout the world, as it was reported as 30.6% in Japan,^38^ 48.8% in Qatar,^39^ 34.7% in the United States,^40^ 33.5% in Turkey,^41^ and 23% in Sweden.^23^ Various statistics on the prevalence of MetS may be due to differences in people's age distribution, demographic characteristics, and lifestyles.

In the primary results of the present study, a significant association between moderate and high MT and the increasing MetS has been found. It was associated with three components of the MetS. According to a study in Finland, MT were related to several components of MetS.^42^ Studies by Zhu in the United States and Hyvärinen stated participants with MT were more likely to have MetS.^43^ In agreement with previous studies,^42^, ^43^ the number of MT was associated with WC, BP, and FBS in this study. A causal relationship may be bidirectional. On the one hand, altered dietary patterns due to tooth MT^44^ or uncontrolled BP and FBS in patients may contribute to tooth loss.^20^ Similarly, the daily brush was related to the WC and HDL‐C. Also, MetS was significantly higher in the case with a lower frequency of brushing per day.^14^ These findings are supported by the results of this study. However, in the presence of demographic and socioeconomic factors, this study failed to observe significant associations.

The findings of this research indicated that individuals with daily flossing, DT, and filled teeth significantly had lower odds of developing MetS. Similar studies reported that the lack of daily flossing was associated with obesity and no dental flossing may increase the likelihood of MetS.^14^ In contrast, another study shows people with DT and FT had a higher prevalence of MetS than those without. Data from 4716 middle‐aged male Japanese indicated decayed teeth were significantly linked to central obesity, high BP, and fasting blood sugar.^9^, ^20^ A study on MetS and DT in Finland revealed no connection between the two conditions.^45^ However, these findings are not supported by the results of the present study, and there is no explanation for these contrasts. Untreated tooth decay can indirectly contribute to MetS due to chronic inflammation and infection in the oral cavity. Periodontitis and untreated DT share common risk factors with MetS, such as poor oral hygiene, unhealthy diet, smoking, and certain demographic factors.^9^, ^20^ It should be noted that there is a direct causal relationship between oral hygiene and long‐term tooth decay.

In the presence of demographic and socioeconomic factors, none of them are related to MetS, and only daily flossing significantly decreases the odds of MetS. Data from previous studies suggest that daily flossing is associated with a lower prevalence of periodontitis^25^ and inflammation which may contribute directly to the development of MetS.^12^ The inflammation from periodontitis spreads throughout the body, causing systemic inflammation. This inflammation disrupts metabolic processes, promotes insulin resistance, and contributes to the development of MetS.^46^ Poor oral health, including untreated tooth decay, can lead to chronic inflammation and infection within the oral cavity. On the other hand, periodontitis and untreated DT share common risk factors with MetS. These risk factors include poor oral hygiene practices, unhealthy dietary habits, smoking, and certain demographic and socioeconomic factors. What is clear is the direct and causal relationship between oral hygiene and tooth decay in the long term.

Adjusted ORs for MetS according to the demographic and socioeconomic factors showed that similarly in other studies, the prevalence of MetS was higher in females than men.^28^ Although a study in India^47^ reported that MetS is higher in men than in women or in Chinese^48^ and the Canadian^22^ revealed that the prevalence of MetS in men and women is equal, in Iran, women are less active, overweight, and obese.^49^ Furthermore, in some countries in the region, the traditional dressing may indirectly contribute to obesity. The traditional long and wide dress for women in Iran especially among Kurdish people may hide the fatness of people and decrease their motivation to lose weight. The traditional dressing makes it difficult to observe the size and shape of a woman's body and even it encourages women to gain more weight.^50^

In the present study, odds of MetS directly increased across age groups. In studies inside and outside Iran, the prevalence of MetS steadily increased with aging.^28^, ^29^ In South Korea,^51^ the incidence of the MetS increased in people with a lower education level. In Swedish, education level was significantly correlated with BP, waist‐to‐hip ratio, HDL‐C level, and serum TG level.^26^ Also, a family history of diabetes was a risk factor for MetS. Previous studies had also found that a history of diabetes is a risk factor for body fatness, Type 2 DM, high BP, lipid abnormalities.^52^, ^53^ Zuo et al. showed that family history of diabetes was independently associated with MetS.^54^ Although, family history represents the ramifications of numerous genetic variables and the family's clustered lifestyle variables^55^ as well, in a cohort study, no association was found.^56^

In the present study, however, the odds of MetS were higher in people who use salt and excessive added sugar intake was inversely related to the MetS. In a study of Iran, salty and sweet increased the incidence of MetS.^57^ A study in Korea revealed that high sodium intake is associated with MetS.^58^ There is strong evidence of the association between the use of salt and high BP/MetS^59^, ^60^ which is supported by the present study. However, inversely in other studies, there is a positive association between sugar intake and MetS.^27^, ^61^ Previous studies had also found that total sugar intake is a risk factor for being overweight^27^ and incidence of type 2 diabetes.^21^ May be due to more adherences to the diet and the recommendations of the nutritionist among people with diabetes or high blood sugar, high BP, and obese people, sugar consumption was reduced and in the present study, no connection was found.

### 4.1 Limitation

The assessment of oral hygiene behavior and other variables relies on self‐reported data, which can introduce recall bias or subjective interpretation. While the study adjusts for sociodemographic characteristics, there may be additional confounding factors that influence both ODH and MetS but were not fully accounted for. It's important to note that due to the cross‐sectional design of this study and the unclear temporal relationship between the variables, the effect size values should be interpreted cautiously.

## 5 CONCLUSION

With considerate this limitation data from present studies suggest that among ODH factors only daily flossing is independently associated with a lower prevalence of MetS. It seems that the sociodemographic characteristics of the participants confounded the relationship between other ODH factors and MetS. Oral health professionals can proactively enhance health awareness and healthcare to prevent MetS.

## AUTHOR CONTRIBUTIONS

**Farhad Moradpour**: Conceptualization; data curation; formal analysis; funding acquisition; investigation; methodology; project administration; writing—original draft; writing—review and editing. **Zahra Karimi**: Investigation; methodology; writing—original draft. **Zeinab Fatemi**: Data curation; investigation; writing—original draft. **Yousef Moradi**: Methodology; writing—original draft. **Mohammad Rastegar Khosravi**: Project administration; writing—review and editing. **Azad Shokri**: Conceptualization; data curation; methodology; supervision; writing—original draft; writing—review and editing. **Mohammad Karimzadeh**: Data curation; methodology.

## CONFLICT OF INTEREST STATEMENT

The authors declare no conflict of interest.

## TRANSPARENCY STATEMENT

The lead author Zahra Karimi, Azad Shokri affirms that this manuscript is an honest, accurate, and transparent account of the study being reported; that no important aspects of the study have been omitted; and that any discrepancies from the study as planned (and, if relevant, registered) have been explained.

## Data Availability

The data sets used and/or analyzed during the current study are available from the corresponding author on reasonable request.

## References

[1] Saklayen MG . The global epidemic of the metabolic syndrome. Curr Hypertens Rep. 2018;20(2):12. 29480368 10.1007/s11906-018-0812-z PMC5866840

[2] Kaur J . A comprehensive review on metabolic syndrome. Cardiol Res Pract. 2014;2014:1‐21. 10.1155/2014/943162 PMC3966331 24711954

[3] Srikanthan P , Daveb JK , Kamdar VV . Cardiovascular disease in South Asians: a burgeoning epidemic. Curr Cardiovasc Risk Rep. 2008;2(3):187‐191.

[4] Shepherd JA , Ng BK , Fan B , et al. Modeling the shape and composition of the human body using dual energy x‐ray absorptiometry images. PLoS One. 2017;12(4):e0175857. 28423041 10.1371/journal.pone.0175857 PMC5397033

[5] Noubiap JJ , Nansseu JR , Lontchi‐Yimagou E , et al. Geographic distribution of metabolic syndrome and its components in the general adult population: a meta‐analysis of global data from 28 million individuals. Diabetes Res Clin Pract. 2022;188:109924. 35584716 10.1016/j.diabres.2022.109924

[6] Ghafuri S , Ghaderi E , Fahami Y , Rajabnia M , Naleini SN . Epidemiologic study of type 2 diabetes mellitus and metabolic syndrome in rural population of kurdistan province, Iran, in 2011–2017. Diabetes Metab Syndr. 2019;13(3):1689‐1697. 31235080 10.1016/j.dsx.2019.03.037

[7] Siegel G , Ermilov E , Knes O , Rodríguez M . Combined lowering of low grade systemic inflammation and insulin resistance in metabolic syndrome patients treated with *Ginkgo biloba* . Atherosclerosis. 2014;237(2):584‐588. 25463092 10.1016/j.atherosclerosis.2014.10.023

[8] Adachi N , Kobayashi Y . One‐year follow‐up study on associations between dental caries, periodontitis, and metabolic syndrome. J Oral Sci. 2020;62(1):52‐56. 31996523 10.2334/josnusd.18-0251

[9] Cao X , Wang D , Zhou J , Yuan H , Chen Z . Relationship between dental caries and metabolic syndrome among 13 998 middle‐aged urban Chinese. J Diabetes. 2017;9(4):378‐385. 27147550 10.1111/1753-0407.12424

[10] Jaramillo A , Lafaurie GI , Millán LV , et al. Association between periodontal disease and plasma levels of cholesterol and triglycerides. Colomb Med. 2013;44(2):80‐86. PMC4002029 24892452

[11] Llambés F . Relationship between diabetes and periodontal infection. World J Diabetes. 2015;6(7):927‐935. 26185600 10.4239/wjd.v6.i7.927 PMC4499526

[12] Si J , Lee C , Ko G . Oral microbiota: microbial biomarkers of metabolic syndrome independent of host genetic factors. Front Cell Infect Microbiol. 2017;7:516. 29326886 10.3389/fcimb.2017.00516 PMC5736563

[13] Broadbent JM , Thomson WM . For debate: problems with the DMF index pertinent to dental caries data analysis. Community Dent Oral Epidemiol. 2005;33(6):400‐409. 16262607 10.1111/j.1600-0528.2005.00259.x PMC1388190

[14] Kim Y‐H , Kim D‐H , Lim KS , et al. Oral health behaviors and metabolic syndrome: the 2008–2010 Korean National Health and Nutrition Examination Survey. Clin Oral Investig. 2014;18:1517‐1524. 10.1007/s00784-013-1112-2 24061606

[15] Shin HS . The number of teeth is inversely associated with metabolic syndrome: a Korean nationwide population‐based study. J Periodontol. 2017;88(9):830‐838. 28452621 10.1902/jop.2017.170089

[16] Moradpour F , Ghaderi E , Moradi G , et al. The Dehgolan Prospective Cohort Study (DehPCS) on non‐communicable diseases in a Kurdish community in the west of Iran. Epidemiol Health. 2021;43:e2021075. 34607397 10.4178/epih.e2021075 PMC8850945

[17] Johnson RK , Appel LJ , Brands M , et al. Dietary sugars intake and cardiovascular health: a scientific statement from the American Heart Association. Circulation. 2009;120(11):1011‐1020. 19704096 10.1161/CIRCULATIONAHA.109.192627

[18] Lu B , Zhang S , Wen J , et al. The new unified international diabetes federation/American heart association/national heart, lung, and blood institute metabolic syndrome definition: does it correlate better with C‐reactive protein in Chinese patients diagnosed with type 2 diabetes? J Int Med Res. 2010;38(6):1923‐1932. 21226995 10.1177/147323001003800605

[19] World Health Organization . Oral Health Surveys: Basic Methods. World Health Organization; 2013.

[20] Ojima M , Amano A , Kurata S . Relationship between decayed teeth and metabolic syndrome: data from 4716 middle‐aged male Japanese employees. J Epidemiol. 2015;25(3):204‐211. 25716056 10.2188/jea.JE20140132 PMC4340997

[21] Papier K , D'este C , Bain C , et al. Consumption of sugar‐sweetened beverages and type 2 diabetes incidence in Thai adults: results from an 8‐year prospective study. Nutr Diabetes. 2017;7(6):283. 10.1038/nutd.2017.27 PMC5519187 28628126

[22] Riediger ND , Clara I . Prevalence of metabolic syndrome in the Canadian adult population. Can Med Assoc J. 2011;183(15):E1127‐E1134. 21911558 10.1503/cmaj.110070 PMC3193129

[23] Roos V , Elmståhl S , Ingelsson E , Sundström J , Ärnlöv J , Lind L . Alterations in multiple lifestyle factors in subjects with the metabolic syndrome independently of obesity. Metab Syndr Relat Disord. 2017;15(3):118‐123. 28339343 10.1089/met.2016.0120

[24] Saberi HR , Moravveji AR , Fakharian E , Kashani MM , Dehdashti AR . Prevalence of metabolic syndrome in bus and truck drivers in Kashan, Iran. Diabetol Metab Syndr. 2011;3:1‐5. 21595922 10.1186/1758-5996-3-8 PMC3117688

[25] Sambunjak D , Nickerson JW , Poklepovic T , et al. Flossing for the management of periodontal diseases and dental caries in adults. Cochrane Database Syst Rev. 2011;(12):CD008829. 22161438 10.1002/14651858.CD008829.pub2

[26] Seidell JC , Cigolini M , Deslypere J‐P , Charzewska J , Ellsinger B‐M , Cruz A . Body fat distribution in relation to physical activity and smoking habits in 38‐year‐old European men. Am J Epidemiol. 1991;133(3):257‐265. 2000843 10.1093/oxfordjournals.aje.a115870

[27] Seo EH , Kim H , Kwon O . Association between total sugar intake and metabolic syndrome in middle‐aged Korean men and women. Nutrients. 2019;11(9):2042. 31480603 10.3390/nu11092042 PMC6769797

[28] Mazloomzadeh S , Rashidi Khazaghi Z , Mousavinasab N . The prevalence of metabolic syndrome in Iran: a systematic review and meta‐analysis. Iran J Publ Health. 2018;47(4):473‐480. PMC5996331 29900131

[29] Farmanfarma KK , Kaykhaei MA , Adineh HA , Mohammadi M , Dabiri S , Ansari‐Moghaddam A . Prevalence of metabolic syndrome in Iran: a meta‐analysis of 69 studies. Diabetes Metab Syndr. 2019;13(1):792‐799. 30641809 10.1016/j.dsx.2018.11.055

[30] Tabatabaei‐Malazy O , Saeedi Moghaddam S , Rezaei N , et al. A nationwide study of metabolic syndrome prevalence in Iran; a comparative analysis of six definitions. PLoS One. 2021;16(3):e0241926. 33657130 10.1371/journal.pone.0241926 PMC7928520

[31] Amirkalali B , Fakhrzadeh H , Sharifi F , et al. Prevalence of metabolic syndrome and its components in the Iranian adult population: a systematic review and meta‐analysis. Iran Red Crescent Med J. 2015;17(12):e24723. 26756015 10.5812/ircmj.24723 PMC4706734

[32] Ahmadi E , Abdollahzad H , Pasdar Y , et al. Relationship between the consumption of milk‐based oils including butter and kermanshah ghee with metabolic syndrome: ravansar non‐communicable disease cohort study. Diabetes Metab Syndr Obes. 2020;13:1519‐1530. 32440181 10.2147/DMSO.S247412 PMC7211326

[33] Kaykhaei M , Hashemi M , Narouie B , et al. Prevalence of metabolic syndrome in adult population from zahedan, southeast Iran. Iran J Publ Health. 2012;41(2):70‐76. PMC3481671 23113137

[34] Yazdanpanah MH , Sayyadipoor S , Hojati SR , Nikmanesh A , Farjam M , Homayounfar R . The association of metabolic syndrome and its components with electrocardiogram parameters and abnormalities among an Iranian rural population: the fasa Persian cohort study. Diabetes Metab Syndr Obes. 2020;13:2975‐2987. 32943893 10.2147/DMSO.S263093 PMC7467662

[35] Kelishadi R , Razaghi EM , Gouya MM , et al. Association of physical activity and the metabolic syndrome in children and adolescents: CASPIAN study. Horm Res. 2007;67(1):46‐52. 17035710 10.1159/000096121

[36] Hajian‐Tilaki K , Heidari B , Firouzjahi A , Bagherzadeh M , Hajian‐Tilaki A , Halalkhor S . Prevalence of metabolic syndrome and the association with socio‐demographic characteristics and physical activity in urban population of Iranian adults: a population‐based study. Diabetes Metab Syndr. 2014;8(3):170‐176. 25220921 10.1016/j.dsx.2014.04.012

[37] Faramarzi E , Somi M , Ostadrahimi A , et al. Association between food insecurity and metabolic syndrome in North West of Iran: Azar cohort study. J Cardiovasc Thorac Res. 2019;11(3):196‐202. 31579459 10.15171/jcvtr.2019.33 PMC6759622

[38] Yamazaki H , Tauchi S , Kimachi M , et al. Association between pancreatic fat and incidence of metabolic syndrome: a 5‐year Japanese cohort study. J Gastroenterol Hepatol. 2018;33(12):2048‐2054. 29697157 10.1111/jgh.14266

[39] Syed MA , Al Nuaimi AS , Latif Zainel AJA , A/Qotba HA . Prevalence of metabolic syndrome in primary health settings in Qatar: a cross sectional study. BMC Public Health. 2020;20:611. 32362284 10.1186/s12889-020-08609-5 PMC7196222

[40] Hirode G , Wong RJ . Trends in the prevalence of metabolic syndrome in the United States, 2011–2016. JAMA. 2020;323(24):2526‐2528. 32573660 10.1001/jama.2020.4501 PMC7312413

[41] Gündogan K , Bayram F , Capak M , et al. Prevalence of metabolic syndrome in the Mediterranean region of Turkey: evaluation of hypertension, diabetes mellitus, obesity, and dyslipidemia. Metab Syndr Relat Disord. 2009;7(5):427‐434. 19754305 10.1089/met.2008.0068

[42] Hyvärinen K , Salminen A , Salomaa V , Pussinen PJ . Systemic exposure to a common periodontal pathogen and missing teeth are associated with metabolic syndrome. Acta Diabetol. 2015;52:179‐182. 24791962 10.1007/s00592-014-0586-y

[43] Zhu Y , Hollis JH . Associations between the number of natural teeth and metabolic syndrome in adults. J Clin Periodontol. 2015;42(2):113‐120. 25581485 10.1111/jcpe.12361

[44] Wakai K , Naito M , Naito T , et al. Tooth loss and intakes of nutrients and foods: a nationwide survey of Japanese dentists. Community Dent Oral Epidemiol. 2010;38(1):43‐49. 19922495 10.1111/j.1600-0528.2009.00512.x

[45] Timonen P , Niskanen M , Suominen‐Taipale L , Jula A , Knuuttila M , Ylöstalo P . Metabolic syndrome, periodontal infection, and dental caries. J Dent Res. 2010;89(10):1068‐1073. 20647498 10.1177/0022034510376542

[46] Srivastava M , Srivastava R , Verma P , Gautam A . Metabolic syndrome and periodontal disease: an overview for physicians. J Family Med Prim Care. 2019;8(11):3492. 31803642 10.4103/jfmpc.jfmpc_866_19 PMC6881921

[47] Misra A , Khurana L . The metabolic syndrome in South Asians: epidemiology, determinants, and prevention. Metab Syndr Relat Disord. 2009;7(6):497‐514. 19900153 10.1089/met.2009.0024

[48] Jiang B , Zheng Y , Chen Y , et al. Age and gender‐specific distribution of metabolic syndrome components in East China: role of hypertriglyceridemia in the SPECT‐China study. Lipids Health Dis. 2018;17(1):92. 29678174 10.1186/s12944-018-0747-z PMC5910574

[49] Ayatollahi SMT , Ghoreshizadeh Z . Prevalence of obesity and overweight among adults in Iran. Obesity reviews. 2010;11(5):335‐337. 20202133 10.1111/j.1467-789X.2010.00725.x

[50] Abdollahi P , Mann T . Eating disorder symptoms and body image concerns in Iran: comparisons between Iranian women in Iran and in America. Int J Eat Disord. 2001;30(3):259‐268. 11746285 10.1002/eat.1083

[51] Lee W‐Y , Jung C‐H , Park J‐S , Rhee E‐J , Kim S‐W . Effects of smoking, alcohol, exercise, education, and family history on the metabolic syndrome as defined by the ATP III. Diabetes Res Clin Pract. 2005;67(1):70‐77. 15620436 10.1016/j.diabres.2004.05.006

[52] Aman AM , Rasyid H , Bakri S , Patellongi IJ . The association between parents history of Type 2 diabetes with metabolic syndrome component and insulin resistance in non‐diabetic young adult male. Acta Med Indones. 2018;50(4):309‐313. 30630995

[53] Hu X , Yu W , Yang L , et al. The association between first‐degree family history of diabetes and metabolic syndrome. Endocrine Practice. 2019;25(7):678‐683. 30865527 10.4158/EP-2018-0543

[54] Zuo H , Shi Z , Hu X , Wu M , Guo Z , Hussain A . Prevalence of metabolic syndrome and factors associated with its components in Chinese adults. Metabolism. 2009;58(8):1102‐1108. 19481771 10.1016/j.metabol.2009.04.008

[55] Zhu H , Chen X , Zhang B , Yang W , Xing X . Family history of diabetes and the effectiveness of lifestyle intervention on insulin secretion and insulin resistance in Chinese individuals with metabolic syndrome. J Diabetes Res. 2021;2021:1‐9. 10.1155/2021/8822702 PMC7803416 33490287

[56] Dunkley AJ , Taub NA , Davies MJ , Stone MA , Khunti K . Is having a family history of type 2 diabetes or cardiovascular disease a predictive factor for metabolic syndrome? Prim Care Diabetes. 2009;3(1):49‐56. 19268647 10.1016/j.pcd.2009.02.002

[57] Asghari G , Yuzbashian E , Mirmiran P , Bahadoran Z , Azizi F . Prediction of metabolic syndrome by a high intake of energy‐dense nutrient‐poor snacks in Iranian children and adolescents. Pediatr Res. 2016;79(5):697‐704. 26717004 10.1038/pr.2015.270

[58] So CH , Jeong HR , Shim YS . Association of the urinary sodium to urinary specific gravity ratio with metabolic syndrome in Korean children and adolescents: The Korea National Health and Nutrition Examination Survey 2010–2013. PLoS One. 2017;12(12):e0189934. 29253859 10.1371/journal.pone.0189934 PMC5734790

[59] Correia‐Costa L , Cosme D , Nogueira‐Silva L , et al. Gender and obesity modify the impact of salt intake on blood pressure in children. Pediatr Nephrol. 2016;31:279‐288. 26420679 10.1007/s00467-015-3210-7

[60] Yang Q , Zhang Z , Kuklina EV , et al. Sodium intake and blood pressure among US children and adolescents. Pediatrics. 2012;130(4):611‐619. 22987869 10.1542/peds.2011-3870 PMC9011362

[61] Sundborn G , Thornley S , Merriman TR , et al. Are liquid sugars different from solid sugar in their ability to cause metabolic syndrome? Obesity. 2019;27(6):879‐887. 31054268 10.1002/oby.22472

